# {2-Hydr­oxy-3-[4-(2-methoxy­ethyl)­phen­oxy]prop­yl}isopropyl­ammonium hemisuccinate

**DOI:** 10.1107/S160053680901856X

**Published:** 2009-05-23

**Authors:** Gianluca Bartolucci, Bruno Bruni, Silvia A. Coran, Massimo Di Vaira

**Affiliations:** a Dipartimento di Scienze Farmaceutiche, Universitá di Firenze, Via U. Schiff 6, I-50019 Sesto Fiorentino, Firenze, Italy; b Dipartimento di Chimica, Universitá di Firenze, Via della Lastruccia 3, I-50019 Sesto Fiorentino, Firenze, Italy

## Abstract

Metoprolol, a widely used adrenoreceptor blocking drug, is commonly administered as the succinate or tartrate salt. The structure of metoprolol succinate, C_15_H_26_NO_3_^+^·0.5C_4_H_4_O_4_^2−^, is characterized by the presence of ribbons in which cations, generated by *N*-protonation of the metoprolol mol­ecules, are hydrogen bonded to succinate anions. The dicarboxylic acid transfers its H atoms to two metoprolol mol­ecules; the asymmetric unit contains one cation and half an anion, the latter possessing twofold rotational symmetry. There are localized nets of O—H⋯O and N—H⋯O hydrogen bonds along a ribbon, within centrosymmetric arrangements formed by pairs of metoprolol cations and pairs of anions, each of the latter contributing with one of its carboxyl groups to the localized net. This arrangement is repeated along the ribbon by the operation of the twofold axis bis­ecting the anion, as well as by the lattice translation.

## Related literature

For general information on the medical applications of metoprolol, see: Benfield *et al.* (1986 ▶); Moses & Borer (1981 ▶); Brogden *et al.* (1977 ▶); Hainer & Sugg (2007 ▶); Ragnarsson *et al.* (1987 ▶); Sandberg *et al.* (1988 ▶).
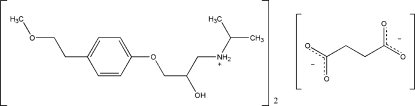


## Experimental

### 

#### Crystal data

C_15_H_26_NO_3_^+^·0.5C_4_H_4_O_4_^2−^
*M* *_r_* = 326.40
Monoclinic, 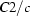

*a* = 26.2630 (4) Å
*b* = 7.9396 (2) Å
*c* = 17.4629 (4) Å
β = 107.348 (2)°
*V* = 3475.68 (13) Å^3^
*Z* = 8
Cu *K*α radiation
μ = 0.75 mm^−1^
*T* = 200 K
0.60 × 0.20 × 0.06 mm

#### Data collection

Oxford Diffraction Xcalibur PX Ultra CCD diffractometer
Absorption correction: multi-scan (*ABSPACK* in *CrysAlisPro RED*; Oxford Diffraction, 2006 ▶) *T*_min_ = 0.732, *T*_max_ = 0.956
22961 measured reflections
3408 independent reflections
3108 reflections with *I* > 2σ(*I*)
*R*_int_ = 0.028

#### Refinement

*R*[*F* ^2^ > 2σ(*F* ^2^)] = 0.045
*wR*(*F* ^2^) = 0.124
*S* = 1.06
3408 reflections
226 parameters
12 restraints
H-atom parameters constrained
Δρ_max_ = 0.21 e Å^−3^
Δρ_min_ = −0.19 e Å^−3^

### 

Data collection: *CrysAlisPro CCD* (Oxford Diffraction, 2006 ▶); cell refinement: *CrysAlisPro CCD*; data reduction: *CrysAlisPro RED* (Oxford Diffraction, 2006 ▶); program(s) used to solve structure: *SIR97* (Altomare *et al.*, 1999 ▶); program(s) used to refine structure: *SHELXL97* (Sheldrick, 2008 ▶); molecular graphics: *ORTEP-3* (Farrugia, 1997 ▶) and *PLATON* (Spek, 2009 ▶); software used to prepare material for publication: *SHELXL97*, *WinGX* (Farrugia, 1999 ▶) and *PARST* (Nardelli, 1995 ▶).

## Supplementary Material

Crystal structure: contains datablocks global, I. DOI: 10.1107/S160053680901856X/pk2162sup1.cif

Structure factors: contains datablocks I. DOI: 10.1107/S160053680901856X/pk2162Isup2.hkl

Additional supplementary materials:  crystallographic information; 3D view; checkCIF report

## Figures and Tables

**Table 1:** Hydrogen-bond geometry (Å, °)

*D*—H⋯*A*	*D*—H	H⋯*A*	*D*⋯*A*	*D*—H⋯*A*
O2—H2O⋯O4^i^	0.84	1.88	2.7231 (15)	179
N—H2N⋯O4^ii^	0.92	1.89	2.7961 (16)	170
N—H1N⋯O5^i^	0.92	1.85	2.7448 (15)	162

Symmetry codes: (i) 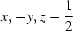; (ii) 
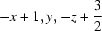
.

**Fig. 1.**
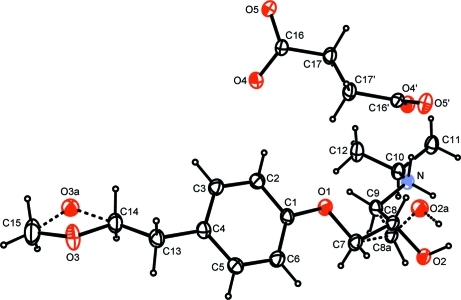
A view of the two ions in the structure of the title compound. The asymmetric unit comprises one metoprolol cation and a half succinate anion, as the latter lies in a site with two–fold rotational symmetry. Primed atoms are related by the operation 1 - x, y, 3/2 - z. Displacement ellipsoids are drawn at the 30% probability level. Minor component disordered atoms are denoted by labels with the trailing letter a and the bonds to which those atoms participate are denoted by dashed lines. For the methyl and methylene groups affected by disorder only the hydrogen atoms belonging to the major fractions are shown for clarity.

**Fig. 2.**
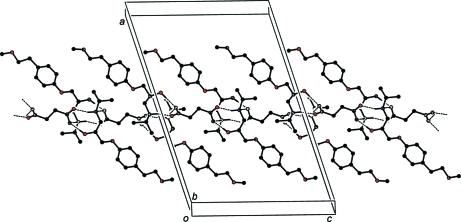
A view, approximately along b, of one of the ribbons, parallel to the c axis direction. Hydrogen bonds are denoted by dashed lines. Only the hydrogen atoms involved in the formation of hydrogen bonds and only the major fractions in the parts affected by disorder are shown.

## supplementary crystallographic information

### Comment

Metoprolol, (±)-1-isopropylamino-3-[4-(2-methoxy-ethyl)-phenoxy]- propan-2-ol, is a β1-selective adrenoreceptor blocking drug, widely used in the treatments of hypertension, angina pectoris and heart failure (Benfield *et al.*, 1986; Moses & Borer, 1981; Brogden *et al.*, 1977). The active substance is provided as metoprolol succinate or tartrate for oral administration, available as extended–release tablets (Hainer & Sugg, 2007; Ragnarsson *et al.*, 1987; Sandberg *et al.*, 1988). Although this drug has been in use for some time, a solid-state structure determination was not yet available. Suitable crystals were obtained for the succinate of the active substance.

The structure of metoprolol succinate consists of cations formed by the N–protonated metoprolol molecule and of succinate dianions, in 2:1 ratio. The asymmetric unit (Fig. 1) contains one metoprolol cation and the symmetry–independent part of the succinate anion, the whole anion possessing two–fold rotational symmetry. Disorder affecting the positions of the ether oxygen O3 and of the hydroxyl oxygen O2 was accounted for. The hydrogen atoms were included in geometrically generated positions, although most of them, including the two ammonium H atoms, could be clearly identified in difference Fourier maps. Consistent with a complete deprotonation of the dicarboxylic acid, the lengths of the two carboxylate C—O bonds are similar. That formed by the O4 atom, which participates in two hydrogen bonds (see below), being only slightly larger (by 0.026 (2) Å) than the other one. In the structure, there are ribbons of hydrogen–bonded ions parallel to the *c* axis (Fig. 2), characterized by the presence of centrosymmetric arrangements where pairs of cations interact with pairs of carboxylate groups belonging to distinct anions. Contiguous arrangements of this type are related to each other along the ribbon by the two–fold rotation axis and two of these contiguous arrangements form the repeat motif in the *c* direction. There are no hydrogen–bond linkages between the ribbons. In detail, the metoprolol cation forms hydrogen bonds to the two O atoms of a carboxylate group through its hydroxyl group (O2···O4^i^ = 2.723 (2) Å, O2—H2O···O4^i^ = 179.4°; symmetry code (i): *x*, - *y*, -1/2 + *z*) and through an ammonium N—H bond (N···O5^i^ = 2.745 (2) Å, N—H1N···O5^i^ = 162.3°). The same metoprolol cation is furthermore linked to the second anion in the centrosymmetric arrangement along the ribbon, *via* the other N—H bond (N···O4^ii^ = 2.796 (2) Å, N—H2N···O4^ii^ = 169.7°; symmetry code (ii): 1 - *x*, *y*, 3/2 - *z*). In this way, each metoprolol cation forms hydrogen bonds to two anions and each succinate anion accepts hydrogen bonds from four cations, through its two carboxylate groups. No carbon atom of the phenyl group deviates by more than 0.005 (1) Å from the best plane through the ring and the O1 and C13 atoms deviate respectively by 0.014 (2) Å and 0.009 (2) Å from it. The dihedral angle between the planes through the two parts of the anion, namely atoms O4, O5, C16 and C17 and the symmetry–related ones, is 82.67 (5)° and the torsion angle through the carbon–atoms backbone of the succinate anion is 179.0 (2)°.

### Experimental

Samples of metoprolol succinate were kindly provided by SIMS (SIMS srl, Reggello Firenze, Italy). Crystals of the compound, suitable for X-ray diffraction analysis, were obtained by slow evaporation from 3:1 methanol:octanol solutions.

### Refinement

Hydrogen atoms were in geometrically generated positions, riding, and the constraint *U(H)* = 1.2*U*_eq_(C,*N*) was applied on the hydrogen temperature factors [*U(H)* = 1.5*U*_eq_(C,*O*) for the H atoms of the methyl and hydroxyl groups]. It appears that a 2.03 Å H···H contact involving the H2' hydrogen of the disordered hydroxyl group, belonging to the fraction with 0.09 occupancy, whose position was (necessarily) geometrically generated, may be ignored, considering that it would be easily released if the hydroxyl O—H bond were allowed to rotate.

A small number (12) of restraints were employed to ensure that the geometry and displacement parameters of the minor-component disordered atoms maintained chemically reasonable values.

### Crystal data

**Table:**

C_15_H_26_NO_3_^+^·0.5C_4_H_4_O_4_^2^^−^	*F*(000) = 1416
*M**_r_* = 326.40	*D*_x_ = 1.248 Mg m^−^^3^
Monoclinic, *C*2/*c*	Cu *K*α radiation, λ = 1.54178 Å
Hall symbol: -C 2yc	Cell parameters from 14350 reflections
*a* = 26.2630 (4) Å	θ = 5.0–72.4°
*b* = 7.9396 (2) Å	µ = 0.75 mm^−^^1^
*c* = 17.4629 (4) Å	*T* = 200 K
β = 107.348 (2)°	Elongated plate, colorless
*V* = 3475.68 (13) Å^3^	0.60 × 0.20 × 0.06 mm
*Z* = 8	

### Data collection

**Table:**

Oxford Diffraction Xcalibur PX Ultra CCD diffractometer	3408 independent reflections
Radiation source: fine-focus sealed tube	3108 reflections with *I* > 2σ(*I*)
Oxford Diffraction Enhance ULTRA assembly	*R*_int_ = 0.028
Detector resolution: 8.1241 pixels mm^-1^	θ_max_ = 72.7°, θ_min_ = 5.3°
ω scans	*h* = −32→32
Absorption correction: multi-scan (ABSPACK in *CrysAlis PRO* RED; Oxford Diffraction, 2006)	*k* = −9→9
*T*_min_ = 0.732, *T*_max_ = 0.956	*l* = −21→18
22961 measured reflections	

### Refinement

**Table:**

Refinement on *F*^2^	Secondary atom site location: difference Fourier map
Least-squares matrix: full	Hydrogen site location: inferred from neighbouring sites
*R*[*F*^2^ > 2σ(*F*^2^)] = 0.045	H-atom parameters constrained
*wR*(*F*^2^) = 0.124	*w* = 1/[σ^2^(*F*_o_^2^) + (0.0619*P*)^2^ + 2.6692*P*] where *P* = (*F*_o_^2^ + 2*F*_c_^2^)/3
*S* = 1.06	(Δ/σ)_max_ < 0.001
3408 reflections	Δρ_max_ = 0.21 e Å^−^^3^
226 parameters	Δρ_min_ = −0.19 e Å^−^^3^
12 restraints	Extinction correction: *SHELXL97* (Sheldrick, 2008), Fc^*^=kFc[1+0.001xFc^2^λ^3^/sin(2θ)]^-1/4^
Primary atom site location: structure-invariant direct methods	Extinction coefficient: 0.00076 (10)

### Special details

**Table:**

Geometry. All e.s.d.'s (except the e.s.d. in the dihedral angle between two l.s. planes) are estimated using the full covariance matrix. The cell e.s.d.'s are taken into account individually in the estimation of e.s.d.'s in distances, angles and torsion angles; correlations between e.s.d.'s in cell parameters are only used when they are defined by crystal symmetry. An approximate (isotropic) treatment of cell e.s.d.'s is used for estimating e.s.d.'s involving l.s. planes.
Refinement. Refinement of *F*^2^ against ALL reflections. The weighted *R*-factor *wR* and goodness of fit *S* are based on *F*^2^, conventional *R*-factors *R* are based on *F*, with *F* set to zero for negative *F*^2^. The threshold expression of *F*^2^ > 2σ(*F*^2^) is used only for calculating *R*-factors(gt) *etc*. and is not relevant to the choice of reflections for refinement. *R*-factors based on *F*^2^ are statistically about twice as large as those based on *F*, and *R*- factors based on ALL data will be even larger.

### Fractional atomic coordinates and isotropic or equivalent isotropic displacement parameters (Å^2^)

**Table:**

	*x*	*y*	*z*	*U*_iso_*/*U*_eq_	Occ. (<1)
C1	0.32938 (5)	0.08129 (18)	0.64968 (8)	0.0315 (3)	
C2	0.34141 (5)	0.01034 (19)	0.72575 (9)	0.0349 (3)	
H2	0.3768	−0.0250	0.7526	0.042*	
C3	0.30174 (6)	−0.00870 (19)	0.76236 (9)	0.0350 (3)	
H3	0.3104	−0.0570	0.8144	0.042*	
C4	0.24918 (5)	0.04133 (18)	0.72460 (8)	0.0321 (3)	
C5	0.23807 (6)	0.1099 (2)	0.64854 (9)	0.0357 (3)	
H5	0.2026	0.1439	0.6213	0.043*	
C6	0.27752 (6)	0.1304 (2)	0.61068 (9)	0.0371 (3)	
H6	0.2689	0.1778	0.5584	0.044*	
O1	0.37140 (4)	0.09661 (14)	0.61815 (6)	0.0375 (3)	
C7	0.36075 (6)	0.1715 (2)	0.54105 (8)	0.0370 (3)	0.907 (3)
H71	0.3487	0.2893	0.5423	0.044*	0.907 (3)
H72	0.3325	0.1081	0.5012	0.044*	0.907 (3)
C8	0.41267 (6)	0.1664 (2)	0.51900 (9)	0.0317 (4)	0.907 (3)
H8	0.4259	0.0477	0.5219	0.038*	0.907 (3)
O2	0.39950 (4)	0.22479 (17)	0.43853 (7)	0.0407 (4)	0.907 (3)
H2O	0.4199	0.1797	0.4155	0.061*	0.907 (3)
C9	0.45477 (6)	0.27692 (19)	0.57594 (9)	0.0349 (3)	0.907 (3)
H91	0.4457	0.3971	0.5641	0.042*	0.907 (3)
H92	0.4553	0.2546	0.6320	0.042*	0.907 (3)
C7'	0.36075 (6)	0.1715 (2)	0.54105 (8)	0.0370 (3)	0.093 (3)
H71'	0.3402	0.2759	0.5413	0.044*	0.093 (3)
H72'	0.3370	0.0940	0.5019	0.044*	0.093 (3)
C8'	0.4070 (4)	0.2166 (17)	0.5093 (7)	0.0317 (4)	0.093 (3)
H8'	0.3968	0.2943	0.4621	0.038*	0.093 (3)
O2'	0.4208 (5)	0.0535 (14)	0.4903 (7)	0.043 (3)*	0.093 (3)
H2'	0.4039	0.0311	0.4424	0.065*	0.093 (3)
C9'	0.45477 (6)	0.27692 (19)	0.57594 (9)	0.0349 (3)	0.093 (3)
H91'	0.4514	0.4002	0.5814	0.042*	0.093 (3)
H92'	0.4532	0.2250	0.6267	0.042*	0.093 (3)
N	0.50840 (4)	0.24135 (15)	0.56677 (7)	0.0311 (3)	
H1N	0.5056	0.2456	0.5130	0.037*	
H2N	0.5178	0.1330	0.5839	0.037*	
C10	0.55297 (6)	0.3574 (2)	0.61095 (9)	0.0367 (3)	
H10	0.5452	0.4731	0.5877	0.044*	
C11	0.60366 (6)	0.2938 (2)	0.59633 (10)	0.0463 (4)	
H111	0.6133	0.1844	0.6226	0.069*	
H112	0.6326	0.3744	0.6184	0.069*	
H113	0.5979	0.2815	0.5385	0.069*	
C12	0.55708 (7)	0.3651 (3)	0.69955 (10)	0.0487 (4)	
H121	0.5605	0.2507	0.7216	0.073*	
H122	0.5249	0.4181	0.7061	0.073*	
H123	0.5885	0.4314	0.7281	0.073*	
C13	0.20539 (6)	0.0195 (2)	0.76350 (9)	0.0375 (3)	
H131	0.1726	0.0738	0.7291	0.045*	
H132	0.1979	−0.1023	0.7658	0.045*	
C14	0.21768 (7)	0.0914 (2)	0.84700 (10)	0.0427 (4)	0.942 (5)
H141	0.2478	0.0302	0.8844	0.051*	0.942 (5)
H142	0.2273	0.2120	0.8472	0.051*	0.942 (5)
O3	0.17048 (7)	0.0715 (2)	0.87015 (10)	0.0595 (6)	0.942 (5)
C15	0.17273 (11)	0.1557 (3)	0.94139 (14)	0.0731 (7)	0.942 (5)
H151	0.2006	0.1053	0.9859	0.110*	0.942 (5)
H152	0.1382	0.1461	0.9519	0.110*	0.942 (5)
H153	0.1809	0.2749	0.9363	0.110*	0.942 (5)
C14'	0.21768 (7)	0.0914 (2)	0.84700 (10)	0.0427 (4)	0.058 (5)
H143	0.2570	0.0876	0.8696	0.051*	0.058 (5)
H144	0.2080	0.2122	0.8398	0.051*	0.058 (5)
O3'	0.1975 (8)	0.0335 (14)	0.9084 (11)	0.038 (6)*	0.058 (5)
C15'	0.17273 (11)	0.1557 (3)	0.94139 (14)	0.0731 (7)	0.058 (5)
H154	0.1401	0.1092	0.9494	0.110*	0.058 (5)
H155	0.1636	0.2525	0.9050	0.110*	0.058 (5)
H156	0.1969	0.1920	0.9931	0.110*	0.058 (5)
C16	0.50084 (5)	−0.18399 (18)	0.86196 (8)	0.0307 (3)	
C17	0.52161 (6)	−0.1855 (2)	0.78974 (8)	0.0380 (3)	
H171	0.5445	−0.0854	0.7923	0.046*	
H172	0.5441	−0.2867	0.7926	0.046*	
O4	0.46519 (4)	−0.07642 (13)	0.86371 (6)	0.0372 (3)	
O5	0.52021 (4)	−0.28659 (14)	0.91717 (6)	0.0430 (3)	

### Atomic displacement parameters (Å^2^)

**Table:**

	*U*^11^	*U*^22^	*U*^33^	*U*^12^	*U*^13^	*U*^23^
C1	0.0271 (6)	0.0390 (7)	0.0318 (7)	−0.0020 (5)	0.0136 (5)	−0.0015 (6)
C2	0.0280 (6)	0.0432 (8)	0.0338 (7)	0.0009 (6)	0.0100 (5)	0.0023 (6)
C3	0.0324 (7)	0.0431 (8)	0.0311 (7)	−0.0010 (6)	0.0117 (5)	0.0030 (6)
C4	0.0303 (7)	0.0363 (7)	0.0324 (7)	−0.0022 (5)	0.0136 (5)	−0.0024 (6)
C5	0.0272 (6)	0.0447 (8)	0.0361 (7)	0.0017 (6)	0.0107 (6)	0.0013 (6)
C6	0.0323 (7)	0.0487 (9)	0.0315 (7)	0.0013 (6)	0.0115 (6)	0.0049 (6)
O1	0.0293 (5)	0.0535 (6)	0.0340 (5)	0.0016 (4)	0.0157 (4)	0.0066 (4)
C7	0.0311 (7)	0.0529 (9)	0.0300 (7)	−0.0016 (6)	0.0136 (6)	0.0029 (6)
C8	0.0310 (7)	0.0402 (11)	0.0265 (7)	0.0013 (7)	0.0125 (6)	0.0035 (7)
O2	0.0367 (6)	0.0593 (8)	0.0300 (6)	0.0071 (5)	0.0157 (5)	0.0061 (5)
C9	0.0323 (7)	0.0409 (8)	0.0359 (8)	−0.0019 (6)	0.0170 (6)	−0.0016 (6)
C7'	0.0311 (7)	0.0529 (9)	0.0300 (7)	−0.0016 (6)	0.0136 (6)	0.0029 (6)
C8'	0.0310 (7)	0.0402 (11)	0.0265 (7)	0.0013 (7)	0.0125 (6)	0.0035 (7)
C9'	0.0323 (7)	0.0409 (8)	0.0359 (8)	−0.0019 (6)	0.0170 (6)	−0.0016 (6)
N	0.0296 (6)	0.0377 (6)	0.0290 (6)	−0.0030 (5)	0.0132 (5)	−0.0003 (5)
C10	0.0352 (7)	0.0433 (8)	0.0342 (7)	−0.0086 (6)	0.0143 (6)	−0.0051 (6)
C11	0.0331 (8)	0.0720 (11)	0.0372 (8)	−0.0087 (7)	0.0158 (6)	−0.0074 (8)
C12	0.0401 (8)	0.0724 (12)	0.0373 (9)	−0.0115 (8)	0.0169 (7)	−0.0167 (8)
C13	0.0323 (7)	0.0452 (8)	0.0395 (8)	−0.0027 (6)	0.0177 (6)	0.0009 (6)
C14	0.0455 (8)	0.0484 (9)	0.0421 (8)	0.0002 (7)	0.0251 (7)	0.0009 (7)
O3	0.0598 (11)	0.0758 (10)	0.0593 (11)	−0.0063 (8)	0.0428 (9)	−0.0093 (8)
C15	0.1075 (18)	0.0667 (13)	0.0690 (14)	0.0119 (13)	0.0630 (14)	0.0016 (11)
C14'	0.0455 (8)	0.0484 (9)	0.0421 (8)	0.0002 (7)	0.0251 (7)	0.0009 (7)
C15'	0.1075 (18)	0.0667 (13)	0.0690 (14)	0.0119 (13)	0.0630 (14)	0.0016 (11)
C16	0.0314 (7)	0.0360 (7)	0.0260 (7)	−0.0034 (5)	0.0106 (5)	−0.0038 (5)
C17	0.0337 (7)	0.0535 (9)	0.0303 (7)	0.0025 (6)	0.0150 (6)	0.0017 (6)
O4	0.0383 (5)	0.0404 (6)	0.0370 (6)	0.0025 (4)	0.0173 (4)	−0.0002 (4)
O5	0.0512 (6)	0.0502 (7)	0.0313 (5)	0.0110 (5)	0.0179 (5)	0.0078 (5)

### Geometric parameters (Å, °)

**Table:**

C1—O1	1.3778 (16)	N—H1N	0.9200
C1—C6	1.385 (2)	N—H2N	0.9200
C1—C2	1.390 (2)	C10—C11	1.515 (2)
C2—C3	1.3834 (19)	C10—C12	1.520 (2)
C2—H2	0.9500	C10—H10	1.0000
C3—C4	1.398 (2)	C11—H111	0.9800
C3—H3	0.9500	C11—H112	0.9800
C4—C5	1.384 (2)	C11—H113	0.9800
C4—C13	1.5099 (18)	C12—H121	0.9800
C5—C6	1.395 (2)	C12—H122	0.9800
C5—H5	0.9500	C12—H123	0.9800
C6—H6	0.9500	C13—C14	1.509 (2)
O1—C7	1.4214 (17)	C13—H131	0.9900
C7—C8	1.5241 (19)	C13—H132	0.9900
C7—H71	0.9900	C14—O3	1.4228 (19)
C7—H72	0.9900	C14—H141	0.9900
C8—O2	1.4210 (19)	C14—H142	0.9900
C8—C9	1.525 (2)	O3—C15	1.398 (2)
C8—H8	1.0000	C15—H151	0.9800
O2—H2O	0.8400	C15—H152	0.9800
C9—N	1.4913 (17)	C15—H153	0.9800
C9—H91	0.9900	C16—O5	1.2487 (17)
C9—H92	0.9900	C16—O4	1.2744 (17)
C8'—O2'	1.410 (9)	C16—C17	1.5161 (18)
C8'—H8'	1.0000	C17—C17^i^	1.508 (3)
O2'—H2'	0.8400	C17—H171	0.9900
N—C10	1.5086 (18)	C17—H172	0.9900
			
O1—C1—C6	124.49 (13)	C10—N—H2N	108.2
O1—C1—C2	115.91 (12)	H1N—N—H2N	107.4
C6—C1—C2	119.60 (12)	N—C10—C11	107.28 (12)
C3—C2—C1	119.88 (13)	N—C10—C12	110.72 (12)
C3—C2—H2	120.1	C11—C10—C12	112.69 (13)
C1—C2—H2	120.1	N—C10—H10	108.7
C2—C3—C4	121.64 (13)	C11—C10—H10	108.7
C2—C3—H3	119.2	C12—C10—H10	108.7
C4—C3—H3	119.2	C10—C11—H111	109.5
C5—C4—C3	117.41 (12)	C10—C11—H112	109.5
C5—C4—C13	120.40 (13)	H111—C11—H112	109.5
C3—C4—C13	122.18 (13)	C10—C11—H113	109.5
C4—C5—C6	121.80 (13)	H111—C11—H113	109.5
C4—C5—H5	119.1	H112—C11—H113	109.5
C6—C5—H5	119.1	C10—C12—H121	109.5
C1—C6—C5	119.66 (13)	C10—C12—H122	109.5
C1—C6—H6	120.2	H121—C12—H122	109.5
C5—C6—H6	120.2	C10—C12—H123	109.5
C1—O1—C7	117.42 (10)	H121—C12—H123	109.5
O1—C7—C8	106.85 (12)	H122—C12—H123	109.5
O1—C7—H71	110.4	C14—C13—C4	114.78 (12)
C8—C7—H71	110.4	C14—C13—H131	108.6
O1—C7—H72	110.4	C4—C13—H131	108.6
C8—C7—H72	110.4	C14—C13—H132	108.6
H71—C7—H72	108.6	C4—C13—H132	108.6
O2—C8—C7	105.59 (12)	H131—C13—H132	107.5
O2—C8—C9	111.85 (13)	O3—C14—C13	106.20 (14)
C7—C8—C9	110.43 (12)	O3—C14—H141	110.5
O2—C8—H8	109.6	C13—C14—H141	110.5
C7—C8—H8	109.6	O3—C14—H142	110.5
C9—C8—H8	109.6	C13—C14—H142	110.5
N—C9—C8	110.19 (12)	H141—C14—H142	108.7
N—C9—H91	109.6	C15—O3—C14	112.98 (17)
C8—C9—H91	109.6	O5—C16—O4	123.48 (12)
N—C9—H92	109.6	O5—C16—C17	118.19 (12)
C8—C9—H92	109.6	O4—C16—C17	118.32 (12)
H91—C9—H92	108.1	C17^i^—C17—C16	113.99 (15)
O2'—C8'—H8'	113.4	C17^i^—C17—H171	108.8
C8'—O2'—H2'	109.5	C16—C17—H171	108.8
C9—N—C10	116.31 (11)	C17^i^—C17—H172	108.8
C9—N—H1N	108.2	C16—C17—H172	108.8
C10—N—H1N	108.2	H171—C17—H172	107.6
C9—N—H2N	108.2		
			
O1—C1—C2—C3	179.49 (13)	O1—C7—C8—O2	173.83 (12)
C6—C1—C2—C3	−0.8 (2)	O1—C7—C8—C9	−65.09 (17)
C1—C2—C3—C4	0.2 (2)	O2—C8—C9—N	−75.80 (16)
C2—C3—C4—C5	0.5 (2)	C7—C8—C9—N	166.91 (12)
C2—C3—C4—C13	179.31 (14)	C8—C9—N—C10	171.96 (12)
C3—C4—C5—C6	−0.6 (2)	C9—N—C10—C11	177.49 (12)
C13—C4—C5—C6	−179.44 (14)	C9—N—C10—C12	54.15 (17)
O1—C1—C6—C5	−179.62 (14)	C5—C4—C13—C14	−128.73 (16)
C2—C1—C6—C5	0.7 (2)	C3—C4—C13—C14	52.5 (2)
C4—C5—C6—C1	0.0 (2)	C4—C13—C14—O3	175.14 (14)
C6—C1—O1—C7	1.5 (2)	C13—C14—O3—C15	−170.64 (17)
C2—C1—O1—C7	−178.87 (13)	O5—C16—C17—C17^i^	132.10 (11)
C1—O1—C7—C8	−177.63 (12)	O4—C16—C17—C17^i^	−49.01 (14)

Symmetry codes: (i) −*x*+1, *y*, −*z*+3/2.

### Hydrogen-bond geometry (Å, °)

**Table:**

*D*—H···*A*	*D*—H	H···*A*	*D*···*A*	*D*—H···*A*
O2—H2O···O4^ii^	0.84	1.88	2.7231 (15)	179
N—H2N···O4^i^	0.92	1.89	2.7961 (16)	170
N—H1N···O5^ii^	0.92	1.85	2.7448 (15)	162

Symmetry codes: (ii) *x*, −*y*, *z*−1/2; (i) −*x*+1, *y*, −*z*+3/2.

## References

[bb1] Altomare, A., Burla, M. C., Camalli, M., Cascarano, G. L., Giacovazzo, C., Guagliardi, A., Moliterni, A. G. G., Polidori, G. & Spagna, R. (1999). *J. Appl. Cryst.* **32**, 115–119.

[bb2] Benfield, P., Clissold, S. P. & Brogden, R. N. (1986). *Drugs*, **31**, 376–429. 10.2165/00003495-198631050-00002 2940080

[bb3] Brogden, R. N., Heel, R. C., Speight, T. M. & Avery, G. S. (1977). *Drugs*, **14**, 321–348. 10.2165/00003495-197714050-00001 201441

[bb4] Farrugia, L. J. (1997). *J. Appl. Cryst.* **30**, 565.

[bb5] Farrugia, L. J. (1999). *J. Appl. Cryst.* **32**, 837–838.

[bb6] Hainer, J. W. & Sugg, J. (2007). *Vasc. Heal. Risk Manag.* **3**, 279–288. PMC2293963 17703635

[bb7] Moses, J. W. & Borer, J. S. (1981). *Dis. Mon.* **27**, 1–61. 10.1016/s0011-5029(81)80014-4 6114824

[bb8] Nardelli, M. (1995). *J. Appl. Cryst.* **28**, 659.

[bb9] Oxford Diffraction (2006). *CrysAlisPro CCD* and *CrysAlisPro RED* (including *ABSPACK*). Oxford Diffraction Ltd, Abingdon, Oxfordshire, England.

[bb10] Ragnarsson, G., Sandberg, A., Jonsson, U. E. & Sjoegren, J. (1987). *Drug Dev. Ind. Pharm.* **13** 1495–1509.

[bb11] Sandberg, A., Blomqvist, I., Jonsson, U. E. & Lundborg, P. (1988). *Eur. J. Clin. Pharmacol.* **33**, S9–14. 10.1007/BF00578406 3371395

[bb12] Sheldrick, G. M. (2008). *Acta Cryst.* A**64**, 112–122. 10.1107/S0108767307043930 18156677

[bb13] Spek, A. L. (2009). *Acta Cryst.* D**65**, 148–155. 10.1107/S090744490804362X PMC2631630 19171970

